# Impact of source, packaging and presence of food safety management system on heavy metals levels in spices and herbs

**DOI:** 10.1371/journal.pone.0307884

**Published:** 2024-08-23

**Authors:** Layale Moussa, Hussein F. Hassan, Ioannis N. Savvaidis, Layal Karam

**Affiliations:** 1 Faculty of Nursing & Health Sciences, Department of Nursing & Health Sciences, Notre Dame University-Louaize, Zouk Mikael, Lebanon; 2 Department of Natural Sciences, School of Arts and Sciences, Lebanese American University, Beirut, Lebanon; 3 Department of Chemistry, University of Ioannina, Ioannina, Greece; 4 Department of Environmental Health Sciences, College of Health Sciences, University of Sharjah, Sharjah, United Arab Emirates; 5 Human Nutrition Department, College of Health Sciences, QU Health, Qatar University, Doha, Qatar; Universidad Autonoma de Chihuahua, MEXICO

## Abstract

Spices and herbs are susceptible to various contaminants, including heavy metals. Our study aimed to quantify the levels of lead (Pb), mercury (Hg), cadmium (As), and cadmium (Cd) in 96 composite samples of 13 herbs and spices frequently consumed in Lebanon. Twenty percent (19/96) and 4% (4/96) of the samples exceeded the permissible levels of Pb and Cd, respectively and all the samples met the permissible levels of As and Hg according to Codex Alimentarius standards. For Pb and Cd, unpackaged samples had the highest levels of unacceptable samples of 31% (8/26) and 8% (2/26), respectively. The samples locally packaged in companies with and without Food Safety Management System (FSMS) had the same levels of unacceptable samples of 12% (3/26) and 4% (1/26) for Pb and Cd, respectively. Imported packaged samples had unacceptable levels of Pb (28% (5/18)) and were acceptable for the three other heavy metals.

## 1. Introduction

Spices and herbs are frequently used as the main components in cooking to provide taste, color, and aroma [1,2]. Moreover, they possess antioxidant, antimicrobial, and anti-inflammatory functions [3]. Recently, the usage of spices and herbs has increased significantly in various regions of the world, mainly North America and Europe [4,5].

The size of the global market for spices and herbs is predicted to exceed USD 25.42 billion by 2029 [6]. Regarding the Lebanese market, the average revenue growth of salt and culinary herbs would be 8.39% from 2023 to 2027 [7]. Spices and herbs are used as necessary seasonings in a vast variety of foods, including pasta, poultry, fish, and meat, as well as salads, sauces, snacks, and desserts. The intake of spices and herbs varies from one country to another. According to Vázquez-Fresno et al., 2019 [8], the countries with the highest average consumption of spices and herbs were Latin America, India, and South Africa, followed by Eastern Asia, the Middle East, Africa, Australia and New Zealand, and then Europe. In Lebanon, the intake was found to be 0.438 kg per person in 2019 and reached 0.706 kg per person in 2020 [9]. However, it is commonly known that a variety of environmental contaminants, including pesticides, heavy metals, and mycotoxins, can affect food originated from plants. These pollutants can be introduced via human activities such as growing, harvesting, and processing. They can enter plants through the soil, water, and air and cannot be eliminated by heating or other physical methods [3,10–13]. Heavy metals, particularly arsenic (As), cadmium (Cd), mercury (Hg) and lead (Pb) are not essential elements for human nutrition [3,4]. They are harmful even at extremely low amounts because the kidneys have difficulty eliminating them [4,14,15]. They may cause a variety of harmful effects on several systems, such as the endocrine and neurological systems, which can lead to cancer and improper development [11,15,16]. A total of 88,285 new cases of cancer were recorded in the national cancer registry in Lebanon over an 8-year period. The number of indexed cancer cases increased from 9,308 to 13,013 during 2008 to 2015 and are set to increase significantly by the year 2025 [17].

High concentrations of Cd and As are primarily caused by chemical fertilizers and pesticides, while Pb is primarily due to vehicle emissions and agriculture [18]. Hg contamination is leached from soil, airborne fallout, and natural sources [19,20]. Consequently, monitoring the presence of heavy metals in agricultural products and their discharge into the environment is crucial due to their negative impact on human health. Governmental agencies and international organizations have established maximum acceptable limits. These limits may differ from one country to another due to varied exposure levels [21]. Codex Alimentarius has standards for dried thyme (CXS_328–2017 dried thyme) and contaminants in food and feed (Codex Alimentarius CXS 193–1995). In Lebanon, there are no particular standards for heavy metals in spices and herbs except the ones established by the Lebanese Standards Institution (LIBNOR) for thyme and thyme mixes (NL 677: 2017 (A)) that are also set by Codex Alimentarius [22].

It has lately been reported in numerous studies that heavy metal toxicity levels in spices and herbs are being monitored. The content levels of Hg, Pb, Cd and As were evaluated in different types of spices and herbs utilized in Republic of Korea [2], Italy [3], and Malaysia [15]. In Lebanon, published studies evaluated the levels of heavy metals in *Origanum syriacum* [23], in thyme-based products [24] and in total diet of Lebanese people [25]. Korfali et al. (2013) determined the contents of several heavy metals in commonly used medicinal herbs and their infusions [26]. Other research evaluated the presence of mycotoxins and microbial contamination in Lebanese herbs and spices [12,27,28].

Nevertheless, no current research has been conducted to assess the levels of heavy metal contamination in common spices and herbs found in the Lebanese market. Thus, the goals of this study were to: (i) assess the occurrence of As, Cd, Pb, and Hg in various kinds of spices and herbs available in Lebanese markets; (ii) evaluate the impact of source, packaging, and presence of Food Safety Management System (FSMS) on the levels of heavy metals in spices and herbs; and (iii) determine whether or not the levels of heavy metals in the spices and herbs meet international standards. By fulfilling these objectives, this study aims to fill the related research gap and provide valuable insights into the potential sources and pathways of contamination of spices and herbs. This knowledge can then be used to inform the development of policies and standards, ultimately leading to enhanced consumer protection.

## 2. Materials and methods

### 2.1 Sampling methodology

Based on previously published research, 13 most consumed dried herbs and spices in Lebanon were chosen [5]. The thirteen types included: black pepper, ground cinnamon, cumin, oregano, paprika, red chili, white pepper, thyme, garlic powder, thyme mix, sesame, sumac, and dried mint. They were evaluated using four categories: samples sold unpackaged (in bulk), imported from France, local brands having Food Safety Management System (FSMS), and local brands not having a FSMS. On the other hand, sumac, sesame, dried mint, and thyme were only assessed for the three remaining categories as there were no imported samples available in the Lebanese markets. The source was identified based on the information found on food packaging, indicating the country in which the spices and herbs were packaged. In order to identify whether industries implement FSMS, we reviewed their packages, websites, and reached out to them via both phone and email. Every kind of spices and dried herbs was gathered from five distinct brands in two sets, separated by three months (June and September 2020) to account for seasonal variability. Samples were collected from local markets and supermarkets with branches located across different regions of Lebanon.

### 2.2. Samples collection

With the exception of sumac, sesame, dried mint, and thyme mix, which were only present locally packed (with or without FSMS) or in bulk due to the lack of an imported option in the market, all spices and dried herbs were found in all four categories. Nine spices/herbs × five brands × four categories x two collection times = 360 samples + 4 spices/herbs × five brands × three categories x two collection periods = 120 samples) were bought from the Lebanese markets, totaling 480 individual samples of spices and dried herbs. In the current study, a composite sampling approach was used based on previously published work [24,29–33]. To create a homogenous representative sample, five distinct brands of each type of spice from each category were combined [5,12,13,26]. As a result, testing was done on 96 composite samples (480/5) (S1 File). The composite sampling approach offers the advantage of saving resources by screening the overall market for the studied spices and herbs. Nevertheless, the contamination concentration arising from each individual sample may be diluted by this sampling technique [12]. The samples were frozen until analysis at the Lebanese Agriculture Research Institute (LARI).

### 2.3. Chemicals, reagents, standards

High analytical grade chemicals were employed. Ultra-pure water was obtained from drinking water by treating it using a BOECO pure (Boeckel, Hamburg, Germany) UV/UF water purification system (>18 MΩ·cm^−1^). All stock solutions of hydrogen peroxide (H2O2), concentrated nitric acid (HNO3), and the metals As, Cd, Pb, and Hg (1000 ± 1 mg/L) were acquired from Sigma-Aldrich, Schnelldorf, Germany. Ultra-pure HNO3 65% (v/v) and ultra-pure water were utilized as cleaning agents, standards preparation, and sample preparation. In order to prepare standard solutions, 1% (v/v) HNO3 nitric acid was made from concentrated acid (65% HNO3 (v/v)).

### 2.4. Extraction and sample preparation

Approximately 0.5g of spices or herbs were precisely weighed into a Teflon container that had been previously cleaned in order to facilitate acid digestion. An acid mix was added to the container, consisting 1 mL of H_2_O_2_ and 7 mL of HNO_3_.The containers were left at room temperature until the bubbles stopped forming. A microwave digestion equipment (Milestone—Ethos Plus American Laboratory Trading, San Diego, United States of America) was used to process the samples. Two stages of mineralization were done at a continuous 1000 W microwave power. Step 1 involved raising the temperature to 200°C in 10 minutes and holding it there for another 10 minutes (step 2). The digested samples were cooled to room temperature before being put into a 25 mL volumetric flask and diluted with 1% (v/v) HNO3 [34]. Every sample underwent triple digestion. The same acid mixture that was used for the samples was utilized to prepare the blank and standards. The working standard solutions for As, Cd, and Pb were then made by diluting the certified standard solution (1000 ± 1 mg L^-1^).

### 2.5. Analysis

#### 2.5.1 Determination of As, Cd, and Pb

The contamination level of As, Cd, Pb, and Hg in the composite samples was evaluated in Lebanese Agriculture Research Institute. As, Cd, and Pb were detected in the spice and herb acid extracts using Atomic Absorption Spectroscopy (AAS thermal M series—Graphite Furnace GF95Z (Zeeman Furnace)Thermo Fisher Scientific, Waltham, Massachusetts, United states of America), as reported by [26,35]. The arsenic, cadmium, and lead hollow-cathode lamp radiation source, as well as an auto sampler with Zeeman-effect background correction, were utilized with a Thermo M series Atomic Absorption Spectrometer. The operating conditions of arsenic, cadmium and lead hollow-cathode were those recommended by the manufacturer. A wavelength of 193.7 nm, 228.8 nm, and 217.0 nm was employed for the determination of As, Cd, and Pb, respectively. The band pass used was 0.5 nm. The current used was 90 mA for both As, Pb and 50 mA for Cd. The auto sampler injected 20 μL of the final solution into the graphite tube. The furnace program for As was 100 ◦C increased by 10◦C/s for 30s, 400 ◦C increased by 150◦C/s for 20s. The furnace program for Cd was 100 ◦C increased by 10◦C/s for 30s, 300 ◦C increased by 150◦C/s for 20s. The furnace program for Pb was 100 ◦C increased by 10◦C/s for 30s, 800 ◦C increased by 150◦C/s for 20s.

#### 2.5.2 Determination of Hg

A Direct Mercury Analyzer DMA 80 (Milestone, Sorisole, Italy) was used to analyze hg [36]. The manufacturer supplied the conditions for the mercury analysis. A quartz furnace was filled with 0.1g of the composite sample after it had been placed in the sample holder (a nickel boat). The air pump was used as combustion and carrier gas. After that, mercury was let out of the amalgamator and transferred to a detector, which monitored the radiation’s absorption from a mercury lamp. The detection of elemental mercury was achieved at 253.7 nm wavelength. The heating program was 60 seconds to reach 200 ◦C (drying step), 105 seconds to reach 650 ◦C (ashing step), 3 seconds to reach 850 ◦C. Each sample was analysed in triplicate.

#### 2.5.3. Quality assurance and quality control

For Hg, Cd, As, and Pb, calibration curve graphs were obtained. Excellent coefficient of correlation (R2 > 0.99) was found in the data. The following formulas were used to determine the limits of quantification (LOQ) and detection (LOD) for As, Cd, and Hg: as stated by [3,37, 38], the LOD is 3 × σ/s and the LOQ is 10 × σ/s. Where "s" is the slope of the calibration curve graph derived for each heavy metal, and "σ" is the standard deviation of ten blank measurements. The LOD and LOQ were 4.10^−5^ and 1,2.10^−4^; 3,6.10^−5^ and 1,2. 10^−4^; 2,62. 10^−4^ and 8.76. 10^−4^; and 2.5.10^−4^ and 8,33. 10^−4^ mg. Kg^-1^ for Hg, Cd, As, and Pb, respectively.

The validity of the analytical procedure was tested on three replicate subsamples of cinnamon by spiking the target heavy metal at three different concentration levels because the certified reference material was not available [34,38–41]. Prior to digestion, solid cinnamon samples were spiked. The samples were mixed thoroughly, prepared and treated following the same procedure and under the same conditions stated in section 2.4 for each heavy metal.

The recovery percentages of the spike were calculated as follows:

$$\%Recovery=\frac{(Cspiked\;sample\;–\;Cunspiked\;sample\;result)\;*\;100}{Cadded}$$

Where C _spiked sample result_ is the Pb, Cd, As, and Hg concentration measured in the samples spiked with standards solutions; C _unspiked sample result_ is the Pb, Cd, As, and Hg concentration measured in the unspiked samples and C_added_ was the known concentration of the heavy metals standards solutions added to the samples [38,42]. A good recovery percentage [38] of As, Cd, Pb, and Hg was achieved and ranged from 90 to 103%. The good recovery rates for all analytes indicated minimal matrix effects from the food samples, thereby limiting measurement uncertainty and contributing to the accuracy and reliability of the analytical method used in this study [43,44]. All samples were analysed in the Lebanese Agricultural Research Institute (LARI) accredited under the ISO/IEC 17025 standard. Routine maintenance and calibration procedures were conducted in accordance with the manufacturer’s guidelines to ensure reliability.

### 2.6. Statistical analysis

IBM SPSS version 22.0 (IBM SPSS statistics, Armonk, NY) was used to conduct the statistical analysis. Every composite sample underwent two collections of analyses and three replicate tests. For every kind of dried herb or spice that fell within each category, the means and standard deviations were determined. The significant difference in mean heavy metal levels across the several categories of dried herbs and spices was evaluated using independent-samples Kruskal-Wallis tests for data which are not normally distributed (Cd, Hg, As) and ANOVA for normally distributed data (Pb). The mean levels for each heavy metal in spices/dried herbs were evaluated as acceptable and unacceptable in reference to international standards. Values were considered to be significantly different if *p*-value < 0.05.

## 3. Results and discussion

The content levels of Pb and Cd exceeded the codex Maximum Permissible Limits (MPL) in 20% (19/96), and 4% (4/96) of the condiments samples, respectively. On the other hand, As and Hg in this study were lower than MPL for all the samples. The mean levels of heavy metals in spices and herbs are shown in S1 Table.

### 3.1. Lead

**Fig 1:**
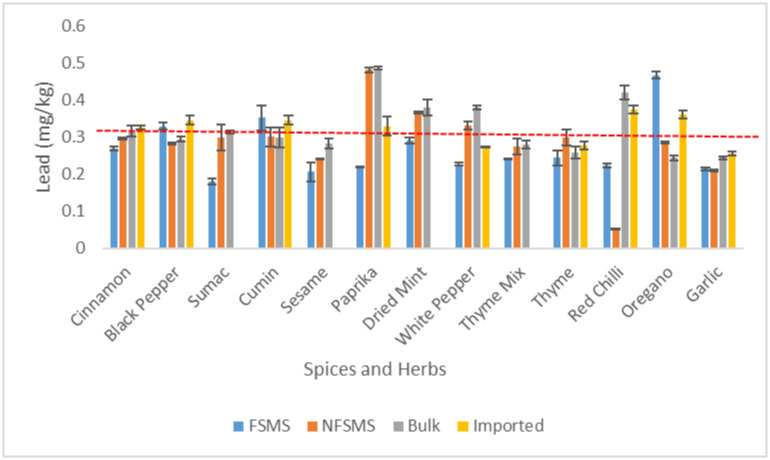
Mean Levels of Pb spices and herbs (in mg/kg) sold unpackaged (bulk), imported and locally packaged with or without implementation of a FSMS (n = 96 composite samples). The line (----) represents the maximum permissible limit (0.3mg.kg^-1^) below which values, were considered acceptable according to Codex Alimentarius (CXS 193–1995). The error bars show the standard deviation.

The range of Pb mean concentrations found in the herbs and seasonings that are sold in the Lebanese market was 0.052 to 0.486 mg.kg^-1^ (Fig 1). Pb levels in these condiments were higher than the MPL (0.3 mg.kg^-1^ set by Codex Alimentarius) in 20% (19/96) of the samples (Table 1). The sample of red chilies that was locally packaged without the use of a food safety management system had the lowest lead levels (0.052 mg/kg) (Fig 1). Unpacked paprika had the greatest lead contamination levels, measuring 0.486 mg.kg-1 (Fig 1), and 75% (6/8) of the paprika samples exceeded the maximum limits. All thyme mix, thyme and garlic powder samples showed unsafe levels of Pb while all sesame samples were acceptable. Pb levels beyond acceptable limits were found in 50% (4/8) of samples of red chili, white pepper, cinnamon, cumin, and oregano; for black pepper, sumac, and dried mint, the unacceptability varied from 25% (2/8) to 33% (2/6) (Table 2). Statistically significant (p-value = 0.0007 < 0.05) mean Pb levels were found among the four categories of spices and dry herbs that were assessed. Similar levels of contamination were observed by Shim et al. (2019), with the average lead content in seasonings and herbs available in Korean markets ranges from 0.039 to 0.972 mg/kg^-1^ [2]. Cinnamon had the largest mean Pb content (0.972 ± 0.893 mg.kg^-1^). Lead concentrations in six different types of condiments commonly used in Europe ranged from < 0.005 to 0.370 μg.g^−1^ for nutmeg and from 0.110 to 0.440 μg.g^−1^ for black pepper [11]. These condiments have lead contents ranging from 0.370 to 1.20. μg.g^−1^. Many previous studies such as Dghaim et al. (2015) and Baig et al. (2019) have demonstrated that the level of Pb exceeded the WHO MPL [10,38]. According to Baig et al. (2019), black cumin seed marketed in the Pakistani market has the highest Pb concentration (47.3–52.7 μg/g) [38]. Dghaim et al. (2015) reported a high concentration of Pb in parsley, chamomile, basil, sage, oregano, and thyme consumed in the United Arab Emirates [10].

**Table 1:** Percentage of permissible and non-permissible levels Pb, Cd, As, and Hg amounts in bulk, imported, and locally packaged spices and herbs with or without FSMS implementation.

Heavy metals	Criterion	Local brands with FSMS	Local brands without FSMS	Imported	Bulk
Lead	% of permissible levels(n/N^a^) % of non-permissible levels (n/N^b^)	88 (23/26) 12 (3/26)	88 (23/26) 12 (3/26)	72 (13/18) 28 (5/18)	69 (18/26) 31 (8/26)
Cadmium	% of permissible levels (n/N^a^) % of non-permissible levels (n/N^b^)	96 (25/26) 4 (1/26)	96 (25/26) 4 (1/26)	100 (18/18) 0 (0/18)	92 (24/26) 8 (2/26)
Arsenic	% of permissible levels (n/N^a^) % of non-permissible levels(n/N^b^)	100 (26/26) 0 (0/26)	100 (26/26) 0 (0/26)	100 (18/18) 0 (0/18)	100 (26/26) 0 (0/26)
Mercury	% of permissible levels (n/N^a^) % of non- permissible levels (n/N^b^)	100 (26/26) 0 (0/26)	100 (26/26) 0 (0/26)	100 (18/18) 0 (0/18)	100 (26/26) 0 (0/26)

^a^ n/N: Number of samples below the permissible levels/total number of samples within the same category.

^b^ n/N: Number of samples above the permissible levels /total number of samples within the same category.

**Table 2:** Percentage of permissible and non-permissible levels of Pb, Cd, As and Hg in different types of spices and herbs.

Spices / herbs	Heavy metals	Lead	Cadmium	Arsenic	Mercury
Cinnamon	% of permissible levels (n/N^a^) % of non-permissible levels (n/N^b^)	50 (4/8) 50 (4/8)	75 (6/8) 25 (2/8)	100 (8/8) 0 (0/8)	100 (8/8) 0 (0/8)
Black pepper	% of permissible levels (n/N^a^) % of non-permissible levels (n/N^b^)	75 (6/8) 25 (2/8)	100 (8/8) 0 (0/8)	100 (8/8) 0 (0/8)	100 (8/8) 0 (0/8)
Sumac	% of permissible levels (n/N^a^) % of non-permissible levels (n/N^b^)	67 (4/6) 33 (2/6)	100 (6/6) 0 (0/6)	100 (6/6) 0 (0/6)	100 (6/6) 0 (0/6)
Cumin	% of permissible levels (n/N^a^) % of non-permissible levels (n/N^b^)	50 (4/8) 50 (4/8)	100 (8/8) 0 (0/8)	100 (8/8) 0 (0/8)	100 (8/8) 0 (0/8)
Sesame	% of permissible levels (n/N^a^) % of non-permissible levels (n/N^b^)	100 (6/6) 0 (0/6)	100 (6/6) 0 (0/6)	100 (6/6) 0 (0/6)	100 (6/6) 0 (0/6)
Paprika	% of permissible levels (n/N^a^) % of non-permissible levels (n/N^b^)	25 (2/8) 75 (6/8)	100 (8/8) 0 (0/8)	100 (8/8) 0 (0/8)	100 (8/8) 0 (0/8)
Dried Mint	% of permissible levels (n/N^a^) % of non-permissible levels (n/N^b^)	67 (4/6) 33 (2/6)	100 (6/6) 0 (0/6)	100 (6/6) 0 (0/6)	100 (6/6) 0 (0/6)
White pepper	% of permissible levels (n/N^a^) % of non-permissible levels (n/N^b^)	50 (4/8) 50 (4/8)	100 (8/8) 0 (0/8)	100 (8/8) 0 (0/8)	100 (8/8) 0 (0/8)
Thyme mix	% of permissible levels (n/N^a^) % of non-permissible levels (n/N^b^)	0 (0/6) 100 (6/6)	83 (5/6) 17 (1/6)	100 (6/6) 0 (0/6)	100 (6/6) 0 (0/6)
Thyme	% of permissible levels (n/N^a^) % of non-permissible levels (n/N^b^)	0 (8/8) 100 (8/8)	100 (8/8) 0 (0/8)	100 (8/8) 0 (0/8)	100 (8/8) 0 (0/8)
Red chili	% of permissible levels (n/N^a^) % of non-permissible levels (n/N^b^)	50 (4/8) 50 (4/8)	88 (7/8) 12 (1/8)	100 (8/8) 0 (0/8)	100 (8/8) 0 (0/8)
Oregano	% of permissible levels (n/N^a^) % of non-permissible levels (n/N^b^)	50 (4/8) 50 (4/8)	100 (8/8) 0 (0/8)	100 (8/8) 0 (0/8)	100 (8/8) 0 (0/8)
Garlic powder	% of permissible levels (n/N^a^) % of non-permissible levels (n/N^b^)	0 (0/8) 100 (8/8)	100 (8/8) 0 (0/8)	100 (8/8) 0 (0/8)	100 (8/8) 0 (0/8)

^a^ n/N: Number of samples below permissible levels samples/total number of samples in all categories.

^b^ n/N: Number of samples above permissible levels samples/total number of samples in all categories.

These findings suggested that the lead (Pb) contamination of spices in the Lebanese market was either lower or comparable to contamination found in other countries. A high concentration of lead may have accumulated as a result of air pollution, the distribution process, and some Pb-containing pesticides used during cultivation, such as lead arsenate [38]. (Baig et al., 2019). On the other hand, the quality of soil is also a factor that attributes to the high level of Pb. This has been demonstrated by a positive association found between soil trace metal concentrations and herb tissue concentrations [23]. Given the toxicity of lead, and its reported accumulation, it cannot be easily eliminated from the food chain through heating or washing fruits and vegetables [15]. This toxic metal has negative consequences on the respiratory, nervous, and renal system [45].

### 3.2. Cadmium

**Fig 2:**
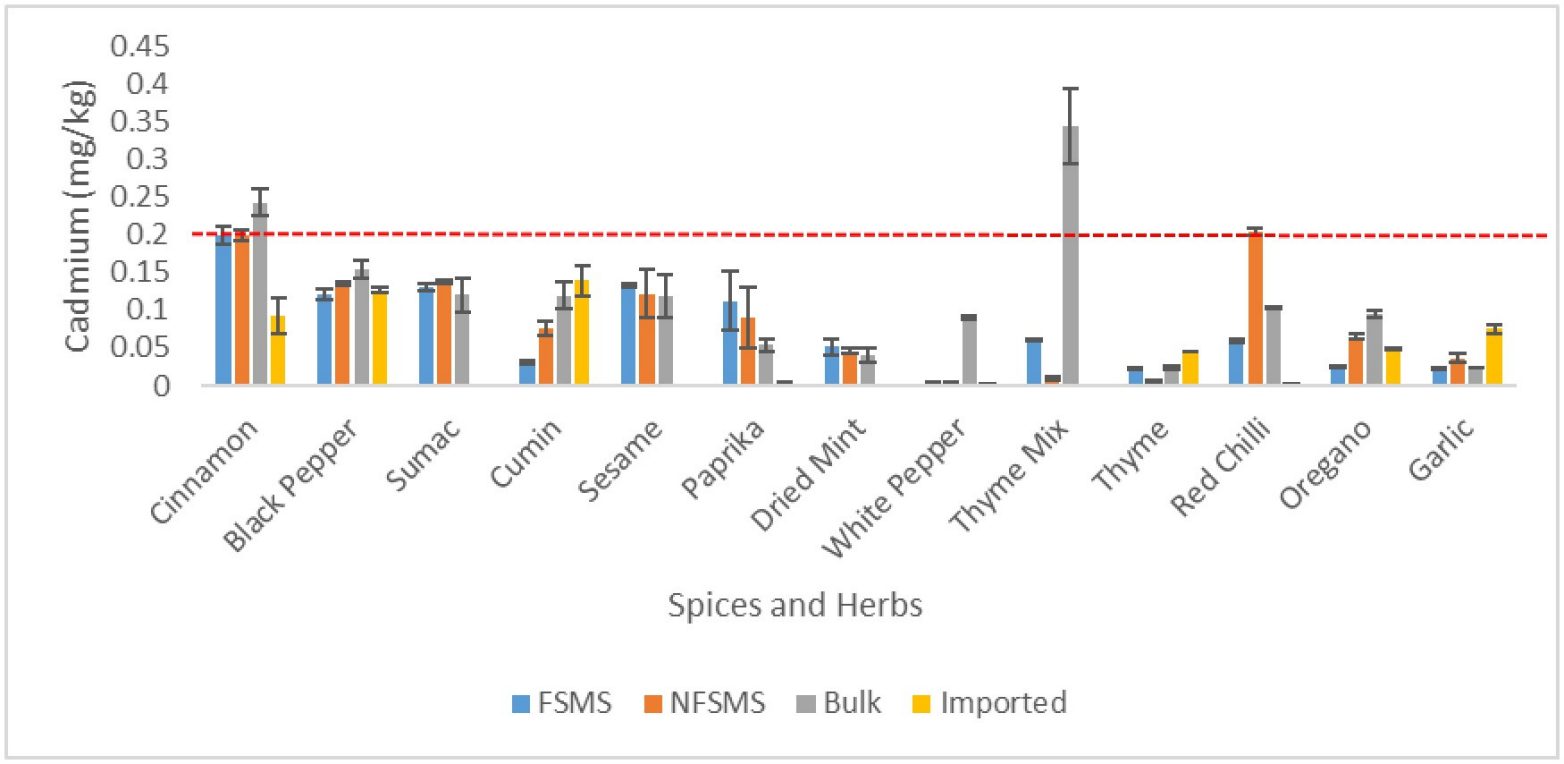
Mean levels of Cd in spices and herbs (in mg/kg) sold unpackaged (bulk), imported and locally packaged with or without implementation of a FSMS (n = 96 composite samples). The line (----) represents the maximum permissible limit (0.2mg.kg-^1^) below which values, were considered acceptable according to Codex Alimentarius (CXS 193–1995). The error bars show the standard deviation.

Spices and herbs had mean concentrations of cadmium ranging from 0.002 to 0.345 mg.kg^-1^. (Fig 2). Cd levels in these condiments were lower than the MPL (0.2 mg.kg^-1^ set by Codex Alimentarius) in 96% (92/96) of the samples (Table 1). In the samples of unpacked cinnamon, locally packaged cinnamon with and without FSMS, and unpacked thyme mix, the amounts of Cd were higher than the permissible limit (0.2 mg.kg-1) established by Codex Alimentarius (Fig 2). In particular, Cd was found at the highest amounts of 0.345 mg.kg^-1^ in unpacked thyme mix (Fig 2). The imported white pepper sample had the lowest content, at 0.002 mg kg^-1^ (Fig 2). Cd levels showed significant difference among spices and herbs categories (*p*-value = 0.0394 < 0.05). Unacceptable amounts of Cd were found in samples of cinnamon, thyme mixture and red chili samples (25% (2/8), 17% (1/6) and 12% (1/8)). All the other condiments met the permissible limits of Cd (Table 2).

Similar to the results of this study (0.044 and 0.088 mg.kg^-1^, respectively), a recent investigation on dry and mixed thyme herbs revealed that the levels of cadmium in Lebanon (0.029 and 0.121 mg.kg^-1^, respectively) were comparable [24].

The Cd levels in spices sold in Hyderabad, Pakistan, was found by Baig et al. (2019), and the concentration range was 0.010–4.70 μg. g-1 [38]. In a study similar to this one, the maximum permitted amount of Cd observed in red chili and cinnamon was exceeded. According to Reinholds et al. (2017), the level of Cd in commercially marketed spices and herbs in Europe is less than 0.2 μg.g^-1^ [11]. The Cd contents of oregano and basil were 0.020 ± 0.010 μg.g-1 and 0.120 ± 0.030 μg.g^-1^, respectively. Thyme was discovered to have a particularly high quantity of Cd (0.054 ± 0.003 mg.kg^−1^), suggesting that the Cd level in Korean thyme was relatively higher than that of European thyme.

Based on study conducted by Korfali et al. (2013), the average amount of Cd in 16 various kinds of medicinal herbs that were sold in the Lebanese market was 0.550 mg.kg-1, which was 1.8 times more than the WHO’s recommended standard of 0.3 mg.kg-1 [26]. The occurrence of Cd in spices and herbs is due to several factors, including the use of polluted irrigation water, wastewater, and fertilizers [26]. The amounts of Cd in agricultural soil can also be influenced by other factors, such as pH, since soil acidification enhances the soil’s capacity to accumulate Cd [46]. A significant factor in the acidity of agricultural soil is the continuous application of fertilizers containing ammonium [47] Consequently, urgent steps for food safety are required, such as cleaning up cadmium contamination in the soil. For instance, the incorporation of both organic and inorganic amendments can successfully reduce the amount of Cadmium that plants absorb. Furthermore, a rise in soil pH has a direct impact on the solubility and mobility of Cd in soil. The amount of Cd that accumulates in plants consequently reduces [48]. A high rate of this metal transfer from soil to fruits and vegetables was predominantly observed [49]. Furthermore, significant quantities of cadmium may be present in the packing materials used to package fresh and prepared foods, which could seep into the food itself [46].

Cadmium in such high concentrations can lead to serious complications like respiratory distress, kidney damage, a drop in bone calcium levels, decreased fertility, and even death [45].

### 3.3. Arsenic

**Fig 3:**
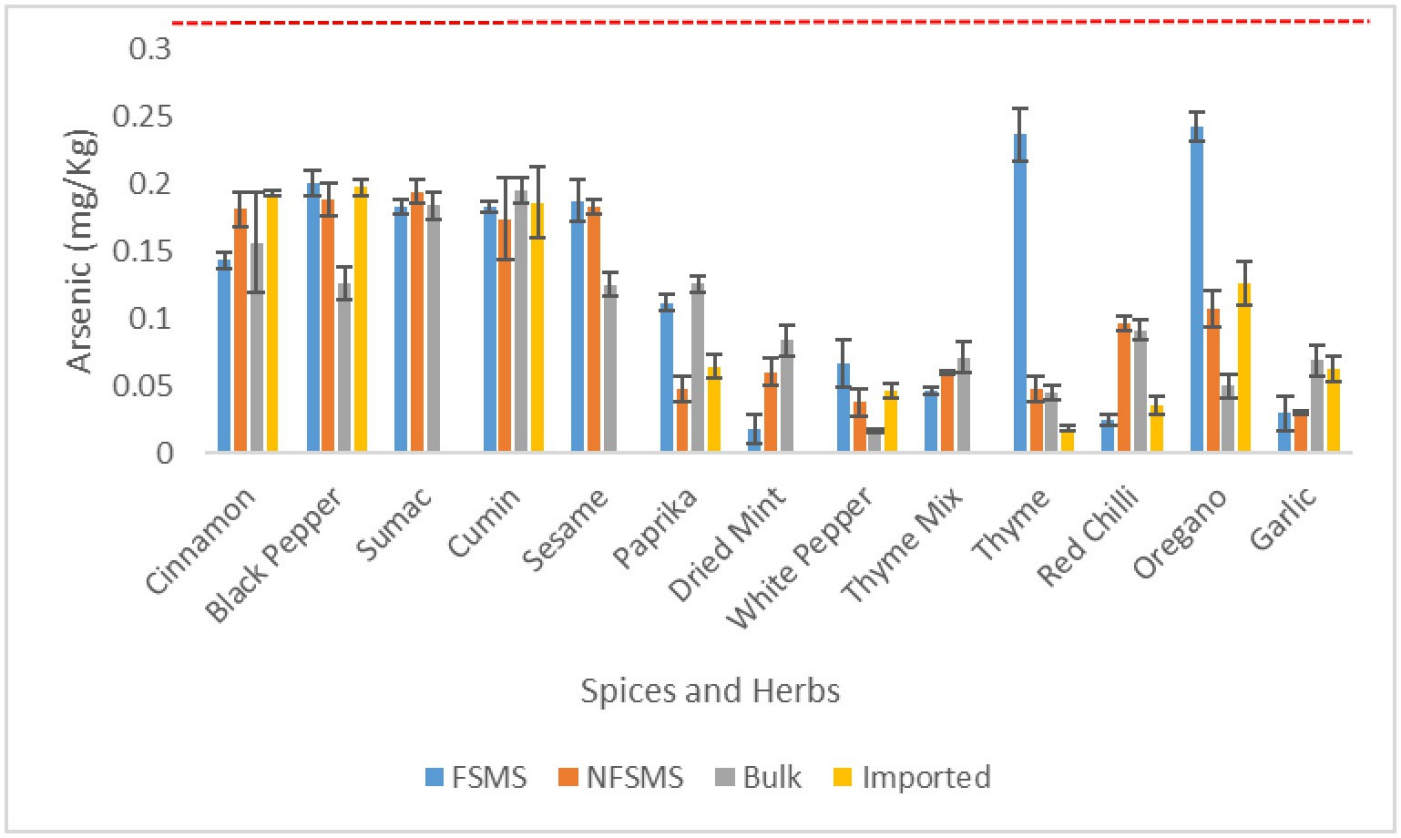
Mean levels of As in spices and herbs (in mg/kg) sold unpackaged (bulk), imported and locally packaged with or without implementation of a FSMS (n = 96 composite samples). The line (----) represents the maximum permissible limit (0.5mg.kg^-1^) below which values, were considered acceptable according to Codex Alimentarius (CXS 193–1995). The error bars show the standard deviation.

Arsenic was found in all the analyzed composite samples of spices and herbs. The range was from 0.017 to 0.242 mg.kg^-1^ (Fig 3). All the spices and herbs composite samples were within the safety limits (Codex Alimentarius 0.5 mg.kg^-1^). There was no statistically significant difference (p-value = 0.5661 > 0.05) in the arsenic level between the categories of herbs and spices. Unpacked white pepper was characterized by the lowest mean in As content (0.017 mg.kg^-1^) and oregano locally packed with FSMS by the highest mean content (0.242 mg.kg^-1^). Similar results were reported in Europe, where the highest mean levels of Arsenic were found in samples of thyme (0.300 ± 0.080 μg.g^−1^) and oregano (0.370 ± 0.090 μg.g^−1^) [11].

The levels of arsenic reported in dried and mixed thyme locally packed without the FSMS and with FSMS respectively (0.048 and 0.046 mg.kg^-1^ respectively) were close to the ones previously reported in dried thyme herb and mixed thyme collected from Lebanese market (0.057 and 0.063 mg.kg^-1^ respectively) [24].

In accordance with Shim et al. (2019), the average As concentration in seasonings sold in Korean markets varied between 0.121 and 0.861 mg.kg^−1^. Thyme (n = 15, 0.861 ± 1.028 mg.kg^−1^) had the highest mean As, while pepper (n = 29, 0.121 ± 0.125 mg.kg^−1^) had the lowest [2].

The amount of As in common spices traded in the Italian market was ascertained by Bua et al. (2016) [3]. They reported ranges from 0.007 to 0.701 mg.kg^−1^, with average As concentrations in ginger, curcuma, and cinnamon of 0.192 ± 0.251 mg.kg^−1^.

Plant concentrations of arsenic are influenced by various factors such as soil composition, water pollution, air pollution, fertilizer use, and other chemical exposure [15]. In addition, the presence of arsenic in food includes both organic and inorganic forms, each with varying levels of toxicity. According to WHO (2010), inorganic As forms like arsenite and arsenate are more toxic to humans and are harmful to most organ systems, with the kidney being the most sensitive. Ingested inorganic arsenic is well absorbed (80–90%) from the gastrointestinal tract, distributed throughout the body, often metabolized via methylation, and primarily excreted in urine [50]. The extent of arsenic poisoning depends on factors like dose, individual susceptibility, and age [50]. Consuming food contaminated with As is known to increase the risk of developing bladder, lung, and skin cancer [51]. Furthermore, frequent consumption of arsenic-containing food can have further long-term health consequences. These can include cardiovascular diseases, skin lesions, disrupted glucose metabolism and diabetes [52]. In contrast, since the body gets rid of organic arsenic compounds quickly, they usually pose less of a threat to human health. (WHO 2019).

### 3.4. Mercury

**Fig 4:**
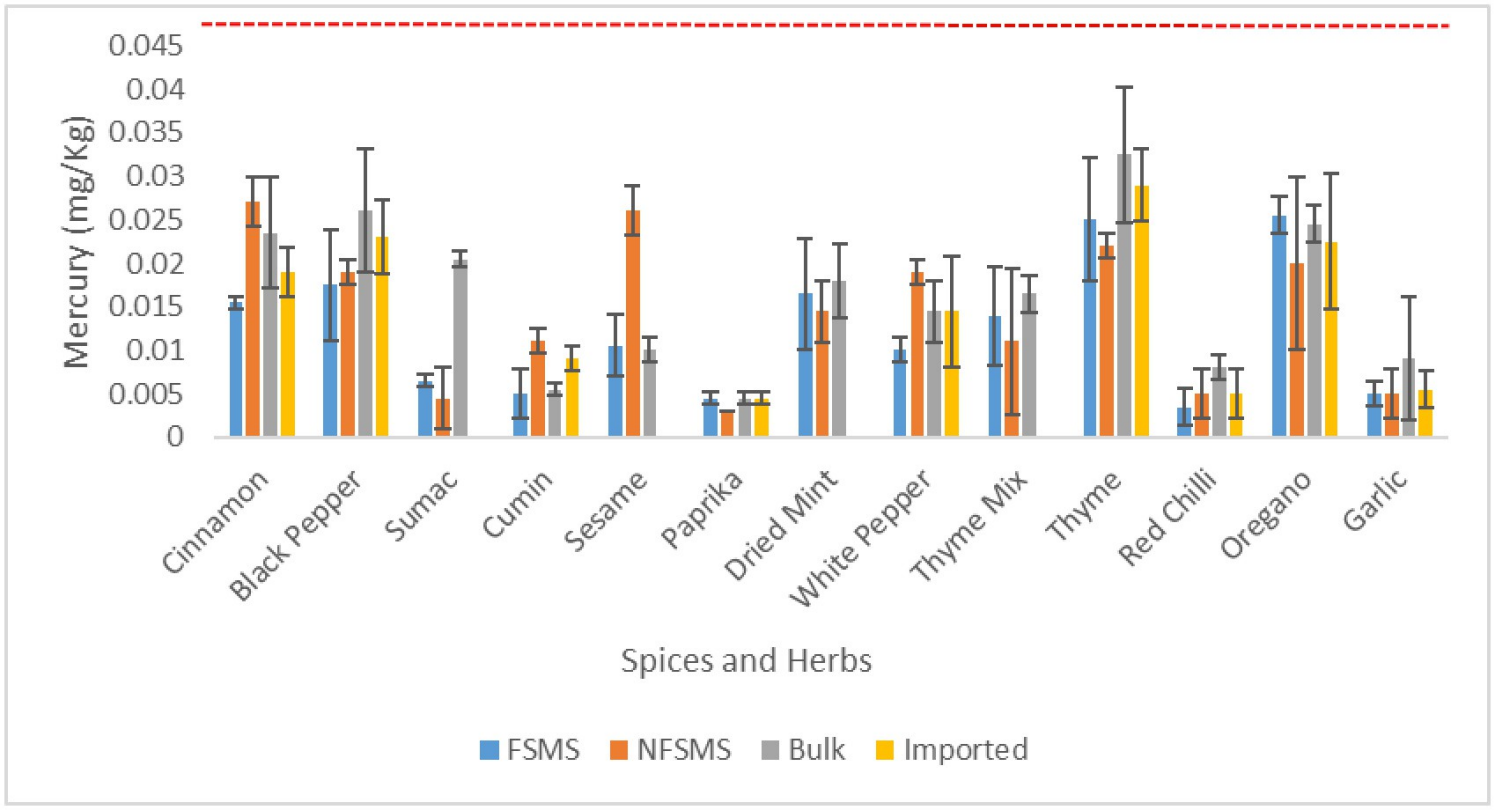
Mean levels of Hg in spices and herbs (in mg/kg) sold unpackaged (bulk), imported and locally packaged with or without implementation of a FSMS (n = 96 composite samples). The line (----) represents the maximum permissible limit (0.1mg.kg^-1^) below which values, were considered acceptable according to Codex Alimentarius (CXS 193–1995). The error bars show the standard deviation.

The mercury level was generally low and did not exhibit a significant variation among spices and herbs categories (*p* = 0.7832 > 0.05). No samples were in the unacceptable range. The mean content of Hg in spices and herbs ranged from 0.003–0.033 mg.kg^-1^ (Fig 4) and none of the analyzed samples contained mercury levels exceeding the permitted limit (0.1 mg.kg^-1^) set by Codex Alimentarius for that metal. The lowest content of mercury was noted at the level of 0.003 mg.kg^-1^ in paprika which is locally packed without FSMS (Fig 4). The highest contamination level with mercury was in unpacked thyme at 0.033 mg.kg^-1^ (Fig 4).

The low levels of mercury in the analyzed samples of spices and herbs were similar to a study done on 20 kinds of spices sold in the Korean markets (2) where the mean content ranged between 0.001 and 0.025 mg.kg^−1^. As reported by Shim et al. (2019), cumin had the lowest quantity of mercury (0.001 ± 0.001 mg.kg−1) while thyme had the largest mean level (0.025 ± 0.026 mg.kg−1) [2]. Spices marketed in the Italian market had Hg concentrations ranging from less than 0.010 to 0.851 mg.kg-1, according to Bua et al. (2016) [3]. Hg concentrations in cinnamon sold in Lebanon were less than those reported for Vietnamese cinnamon supplied in Italy [3]. Malaysian herbs have greater range levels of mercury (0.060 to 0.520 mg.kg−1), as stated by Nordin and Selamat (2013) [13]. Hence, the amount of mercury present in regularly consumed spices and herbs in the Lebanese market is either comparable to or less than that of other nations. All of the samples of oregano available in Lebanese markets had a mercury content that was in good accordance with findings published by Storelli (2014) and Shim et al. (2019) [2,35]. Several environmental factors related to the production or cultivation area can typically be linked to the heavy metal content [3].

The activities of several industries, including the pharmaceutical, agricultural, and caustic soda producing sectors, release mercury into the environment [53]. Mercury toxicity varies depending on its chemical form. It exists in elemental, inorganic, and organic forms, with organic mercury, particularly methylmercury, posing the highest risk in terms of bioaccumulation and associated health hazards [3,46,54]. This form of mercury is mainly found in marine organisms and consuming seafood with elevated mercury levels on a regular and long-term basis carries a hazardous danger [44]. High exposure levels to mercury can damage the brain and kidneys, which makes them particularly dangerous for pregnant women [55,56]. It also has a negative effect on the child’s development during pregnancy and the first few years of life [46].

Among all measured hazardous elements, the highest proportion of unacceptability was reported in Pb followed by Cd. Nevertheless, all the types of dried herbs and spices had acceptable levels of As and Hg. In general, the contamination among spices and herbs was more related to the type of heavy of metals and types of spices and herbs rather than the source or country of packing. This trend could be attributed to the fact that a majority of the world’s spices and herbs are predominantly cultivated in key countries such as India (1.6 million tons), China (99,000 tons), Bangladesh (48,000 tons), Pakistan (45,000 tons), and Nepal (15,000 tons), before being subsequently distributed to various corners of the globe [57]. Consequently, the contamination levels appear to be associated with various farming methods, the particular pesticides used to cultivate different herbs and spices, and the environmental conditions that are in place. Soil acidity and insufficient macronutrient availability are primary factors associated with the accumulation of heavy metals [58]. The interaction of soil contaminants with plant roots leads to the buildup of heavy metals in ecosystems [58]. Moreover, no difference in the level of contamination in Pb and Cd was detected between samples locally packed with and without FSMS. This could be due to the fact that not all organizations view heavy metals as serious hazards to be addressed as part of their FSMS in contrast to microbiological hazards, which are regularly monitored in condiments [28]. Since the existence of trace elements cannot be eliminated during the preparation and packaging of spices and herbs, the quality of the received raw materials is likely the main contributor to the contamination levels in the samples. For this reason, suppliers’ quality assurance programs should be enforced and implemented including selection of approved suppliers and request of certificates of analysis indicating the levels of target heavy metals in their products. Additionally, it’s important to note that the samples that were bought in bulk or without packaging had the highest levels of contamination (31% (8/26) for Pb and 8% (2/26) for Cd. Packaging materials can present protective barrier that prevents environmental contaminants. However, this is only relevant when approved food grade materials that do not leach heavy metals to food are used.

In summary, a variety of factors, including harvesting location sites, country of origin, soil contamination, irrigation water, environmental pollution, good agricultural practices and lack of inspection efforts in companies and countries. are linked to the contamination of spices and herbs with heavy metals. This highlights the importance of farm to fork approach and of routine monitoring tests for toxic metals in spices and herbs. Establishing traceability systems, educating farmers and suppliers quality assurance can help prevent contamination at the source. Regulatory enforcement and scientific based policies can also play a crucial role in ensuring the safety of spices for consumers. It is important to note that the current state of affairs in Lebanon makes it difficult to control the concentrations of heavy metals in spices and herbs. For example, at the ports of entry, the local authorities do not screen for harmful components. Moreover, the borders are open for smuggling with low monitoring by authorities. Furthermore, the Lebanese crisis influenced the quality of the imported product such as bringing in cheap brands.Additionally, the public sector strike limits the enforcement of regulations and product quality assessments. The widespread practice of irrigating crops with sewage raises concerns about public health due to the potential for chemical and microbiological contamination [28].

The consumption and addition of spices and herbs to food is a common practice in Lebanese household. Although some herbs and spices are safe to use in moderation, adding them to nearly every meal may lead to heavy metals accumulation in human organs, which can cause a variety of health issues. It is also noteworthy that some herbs like thyme can contribute up to 35% of the weight of some types of thyme-based items [5,12].

## 4. Conclusions

The current study permitted to assess the occurrence of heavy metals and the impact of packaging practices, source and implementation of FSMS on the compliance levels in spices and herbs commonly commercialized in Lebanon. More restrictive measures should be implemented to control exposure to Pb and Cd through the consumption of those condiments.

Our results highlight the need of efficiently controlling heavy metal content in spices and herbs as a crucial part of comprehensive food safety management. Regular testing for the presence of heavy metals in herbs and spices is necessary to ensure safety throughout the entire production process. Furthermore, studies performing speciation analysis for each metal should be conducted and a risk assessment should be carried out to determine any potential negative health impacts on consumers. Furthermore, maintaining safe levels of hazardous metal content in plants requires strict adherence to and implementation of Good Agricultural Practices. Further recommendations include conducting risk assessments to account for the amount of spices and herbs consumed in the diet, monitoring contamination trends over time, evaluating the impact of supply chain management, and enacting laws to regulate the concentrations of heavy metals in dried herbs and spices. The composite sampling approach used in this study has both limitations and advantages. While it allowed for the screening of a larger number of samples and provided an overall assessment of the status of spices and herbs in the Lebanese market, it may have diluted the contamination levels present in individual samples and can compromise the reproducibility of results. To overcome this study limitation, future research should focus on testing individual samples to gain a more precise understanding of contamination levels. Moreover, it is crucial to prioritize the implementation of risk-based local standards, establish maximum acceptable levels for contaminants, and develop regulations tailored to the country’s consumption habits and dietary patterns.

## Supporting information

S1 File Sampling methodology, identification and source of samples. (DOCX)

S2 File (XLSX)

S1 Table Mean levels of heavy metals (mg/kg) ± Standard Deviation (SD) in spices and herbs. (DOCX)
