# Supplementary material for: Impact of source, packaging and presence of food safety management system on heavy metals levels in spices and herbs
Source: PLoS One. 2024 Aug 23;19(8):e0307884. doi: 10.1371/journal.pone.0307884 (PMC11343411; doi:10.1371/journal.pone.0307884)

**Impact of source, packaging and presence of food safety management system on heavy metals levels in spices and herbs**

Layale Moussa^1^, Hussein F. Hassan^2^, Ioannis N. Savvaidis^3,4^, Layal Karam^5*^

^1^Department of Nursing & Health Sciences, Faculty of Nursing & Health Sciences, Notre Dame University-Louaize, P.O. Box 72, Zouk Mikael, Lebanon,

^2^Department of Natural Sciences, School of Arts and Sciences, Lebanese American University, P.O. Box 13-5053, Beirut 1102-2801, Lebanon

^3^ Department of Chemistry, University of Ioannina, Ioannina 45110, Greece

^4^ Department of Environmental Health Sciences, College of Health Sciences, University of Sharjah, P. O. Box 27272 Sharjah, United Arab Emirates.

^5^Human Nutrition Department, College of Health Sciences, QU Health, Qatar University, P.O. Box 2713, Doha, Qatar

*Correspondence: lkaram@qu.edu.qa

**S1 File. Sampling methodology, identification and source of samples**

**Step 1:** Selection of 13 types of spices and herbs most consumed in Lebanon

**Step 2:** Samples collection

**Step 3:** Preparation of the composite samples

**Step 1:** Selection of 13 types of spices and herbs most consumed in Lebanon

An online survey was carried out including 60 types of spices and herbs commercialized in Lebanon.

*Table 1. List of 60 types of spices and herbs commercialized in Lebanon (El. Darra et al., 2018)*

| Single spices | Mixture spices | Single herbs | Mixture herbs |
| --- | --- | --- | --- |
| All spice | Biryani spices | Bay leaves | Extra Thyme |
| Anise | Chicken spices | Basil | Mix herbs |
| Black pepper | Chicken shawarma spices | Chamomile flowers | Zaatar Halaby |
| Caraway | Curry powder | Corn silk |  |
| Cardamom seed | Falafel spices | Hibiscus |  |
| Cinnamon | Four spices | Marjoram |  |
| Cloves | Francisco spices | Mint dried |  |
| Coriander seeds | Hamburger spices | Molokia |  |
| Cumin | Kasbeh spices | Oregano |  |
| Fennel | Kefta spices | Parsley dried |  |
| Fenugreek seeds | Kibby spices | Primrose |  |
| Garlic powder | Moghrabieh spices | Rosemary |  |
| Ginger | Red tawouk spices | Safflower |  |
| Nutmeg seeds | Rice spices | Saffron |  |
| Onion powder | Sausage spices | Sage |  |
| Paprika | Seven spices | Thyme flower |  |
| Red Chilli | Steak black pepper | Thyme grinded |  |
| Sesame | Ten spices | Violets |  |
| Sumac |  |  |  |
| Turmeric |  |  |  |
| White pepper |  |  |  |

The survey allowed the selection of 13 types of spices and herbs most consumed in Lebanon based on previously published study (Karam et al., 2021).

*
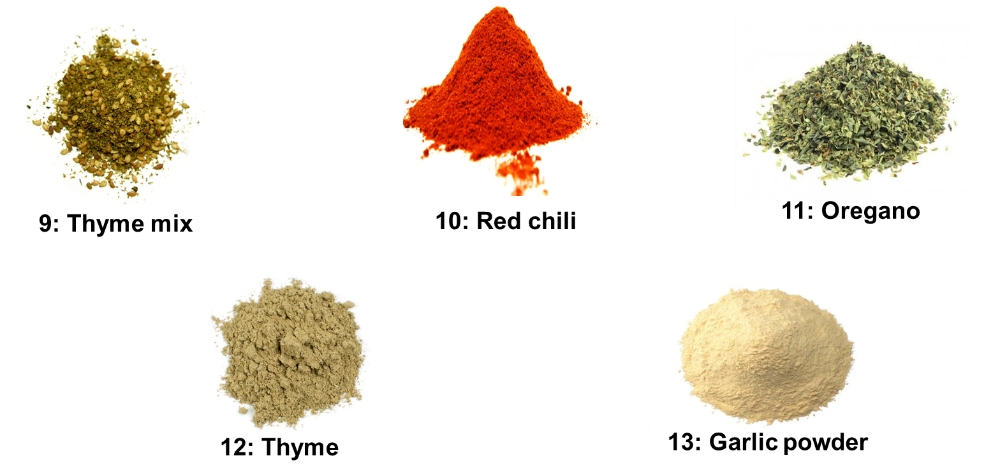

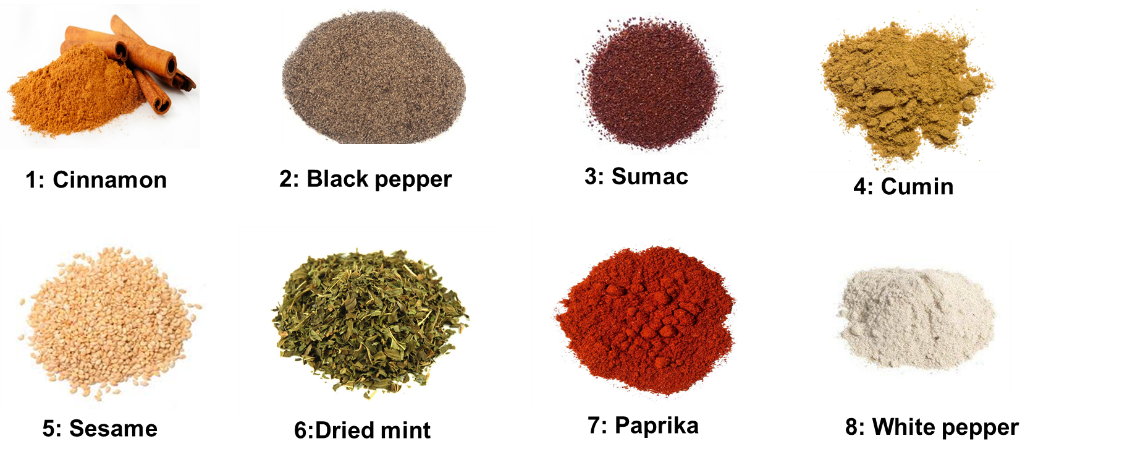
*

*Fig. 1. 13 types of most consumed dried herbs and spices in Lebanon*

**Step 2:** Samples collection

Spices and herbs samples were purchased from four categories: sold unpackaged (in bulk), imported from France, local brands having Food Safety Management System (FSMS), and local brands not having a FSMS (Karam et al., 2021).


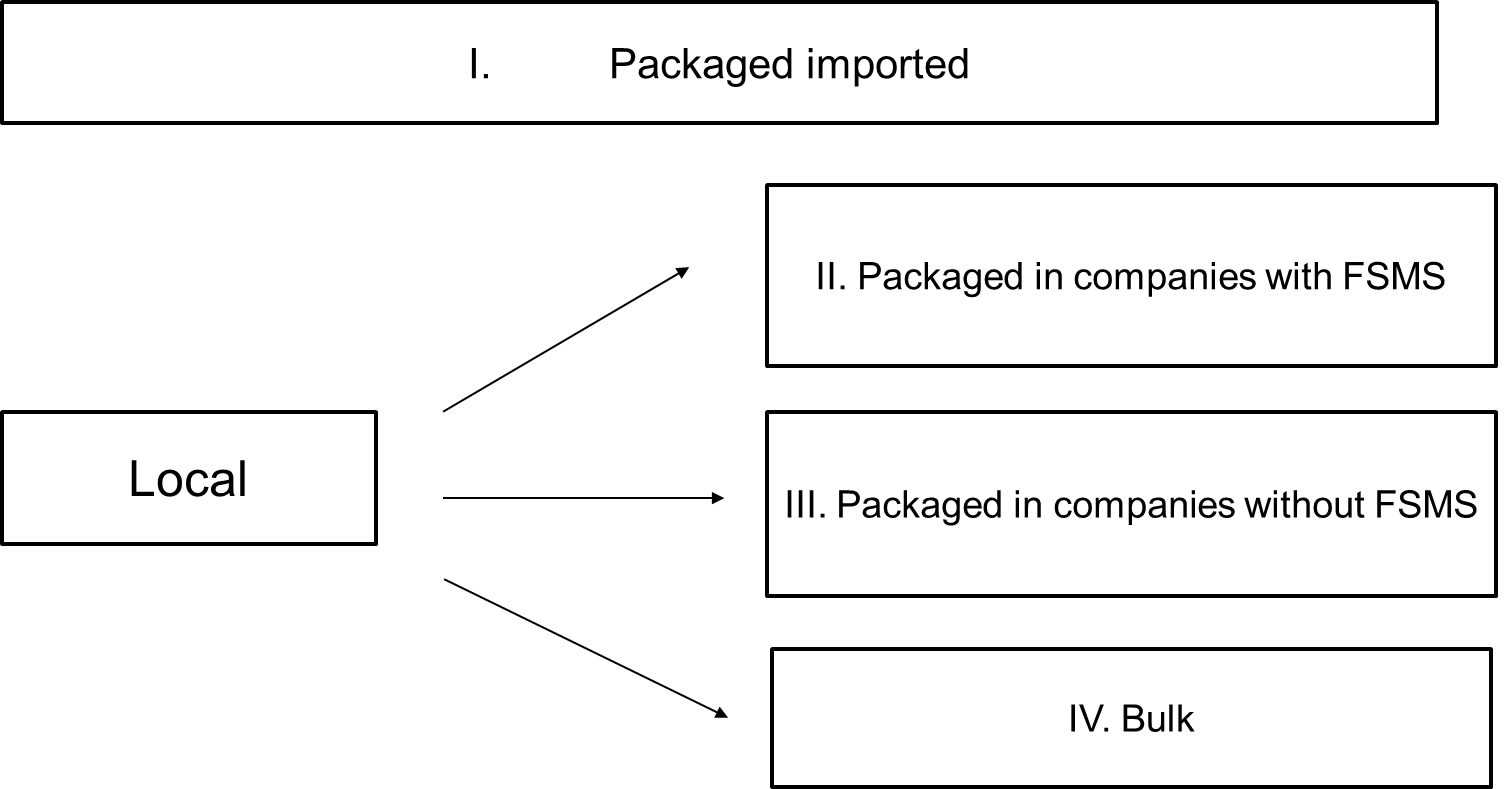


A total of 480 samples of spices and dried herbs were collected at two complete sets of different production dates at three-months interval.

**
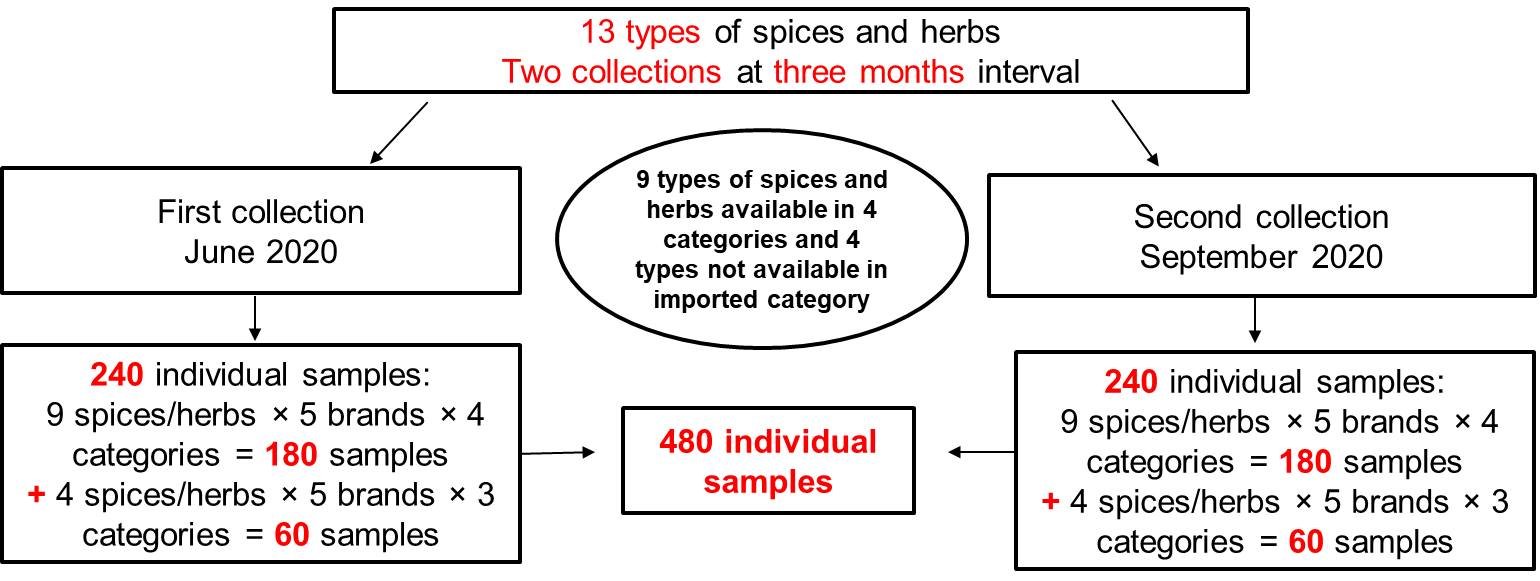
**

**Step 3:** Preparation of the composite samples

A composite sampling approach was applied. Every type of spices and dried herbs was gathered from five distinct common brands collected from local markets and supermarkets with branches located across different regions of Lebanon (Karam et al., 2021). The five individual brands of each sample type (20 g each) were mixed in one sample (100 g) before analysis.


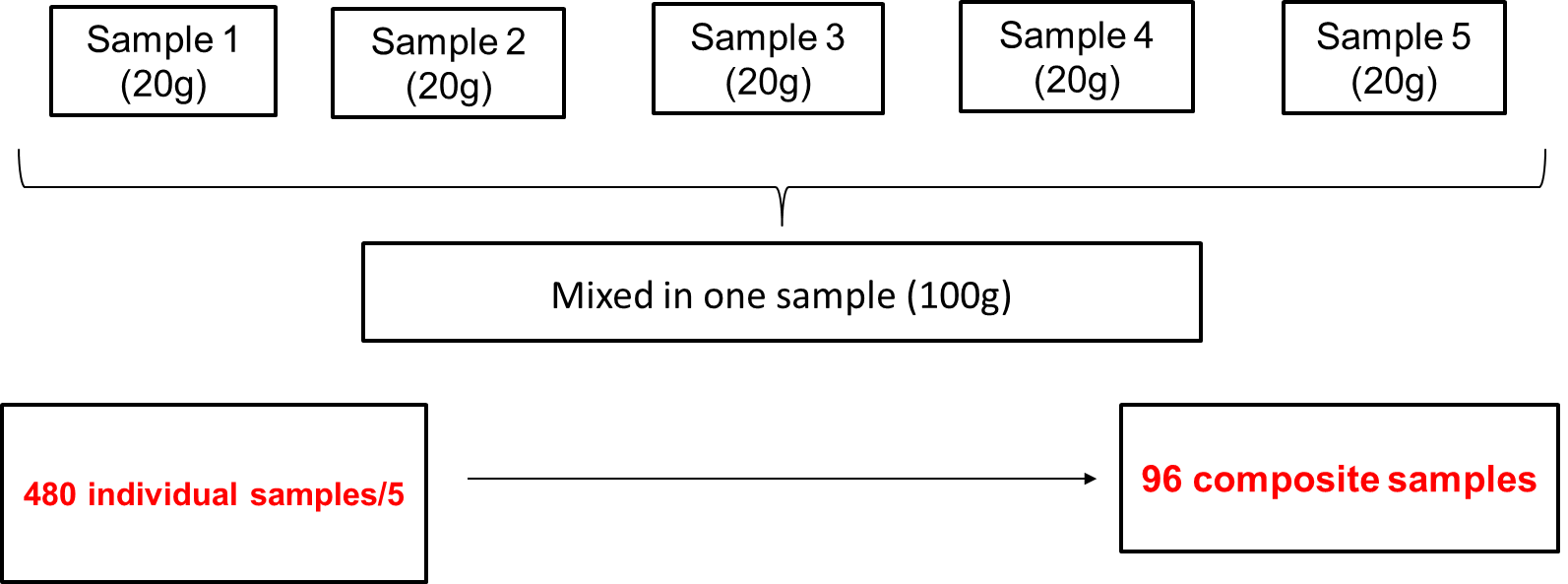

Supplement: S1 File — (DOCX) [file pone.0307884.s001.docx]
