# Supplementary material for: Impact of source, packaging and presence of food safety management system on heavy metals levels in spices and herbs
Source: PLoS One. 2024 Aug 23;19(8):e0307884. doi: 10.1371/journal.pone.0307884 (PMC11343411; doi:10.1371/journal.pone.0307884)
Supplement: S1 Table — (DOCX) [file pone.0307884.s003.docx]

**Impact of source, packaging and presence of food safety management system on heavy metals levels in spices and herbs**

Layale Moussa^1^, Hussein F. Hassan^2^, Ioannis N. Savvaidis^3,4^, Layal Karam^5*^

^1^Department of Nursing & Health Sciences, Faculty of Nursing & Health Sciences, Notre Dame University-Louaize, P.O. Box 72, Zouk Mikael, Lebanon,

^2^Department of Natural Sciences, School of Arts and Sciences, Lebanese American University, P.O. Box 13-5053, Beirut 1102-2801, Lebanon

^3^ Department of Chemistry, University of Ioannina, Ioannina 45110, Greece

^4^ Department of Environmental Health Sciences, College of Health Sciences, University of Sharjah, P. O. Box 27272 Sharjah, United Arab Emirates.

^5^Human Nutrition Department, College of Health Sciences, QU Health, Qatar University, P.O. Box 2713, Doha, Qatar

*Correspondence: [lkaram@qu.edu.qa](mailto:lkaram@qu.edu.qa)

**S1 Table. Mean levels of heavy metals (mg/kg) ± Standard Deviation (SD) in spices and herbs.**

| Spices and Herbs | Categories | Pb | Mean ±SD | Cd | Mean ±SD | As | Mean ±SD | Hg | Mean ±SD |
| --- | --- | --- | --- | --- | --- | --- | --- | --- | --- |
| Cinnamon | FSMS^a^ | 0.265 | 0.269±0.006 | 0.19 | 0.1985±0.012 | 0.147 | 0.143±0.006 | 0.016 | 0.0155±0.0007 |
|  | FSMS^b^ | 0.273 |  | 0.207 |  | 0.139 |  | 0.015 |  |
|  | NFSMS^a^ | 0.299 | 0.297±0.004 | 0.194 | 0.1995±0.0078 | 0.19 | 0.181±0.013 | 0.029 | 0.027±0.003 |
|  | NFSMS^b^ | 0.294 |  | 0.205 |  | 0.172 |  | 0.025 |  |
|  | Bulk^a^ | 0.307 | 0.318±0.015 | 0.256 | 0.243±0.0184 | 0.13 | 0.156±0.037 | 0.028 | 0.0235±0.0064 |
|  | Bulk^b^ | 0.328 |  | 0.23 |  | 0.182 |  | 0.019 |  |
|  | Imported^a^ | 0.323 | 0.326±0.004 | 0.11 | 0.093±0.024 | 0.194 | 0.193±0.002 | 0.017 | 0.019±0.003 |
|  | Imported^b^ | 0.329 |  | 0.076 |  | 0.191 |  | 0.021 |  |
| Black Pepper | FSMS^a^ | 0.324 | 0.33±0.008 | 0.126 | 0.121±0.007 | 0.207 | 0.2±0.01 | 0.013 | 0.175±0.0064 |
|  | FSMS^b^ | 0.336 |  | 0.116 |  | 0.193 |  | 0.022 |  |
|  | NFSMS^a^ | 0.282 | 0.284±0.003 | 0.137 | 0.136±0.002 | 0.197 | 0.189±0.012 | 0.02 | 0.019±0.0014 |
|  | NFSMS^b^ | 0.286 |  | 0.134 |  | 0.18 |  | 0.018 |  |
|  | Bulk^a^ | 0.299 | 0.295±0.006 | 0.145 | 0.154±0.012 | 0.135 | 0.126±0.013 | 0.021 | 0.026±0.0071 |
|  | Bulk^b^ | 0.29 |  | 0.162 |  | 0.117 |  | 0.031 |  |
|  | Imported^a^ | 0.338 | 0.346±0.011 | 0.129 | 0.127±0.003 | 0.201 | 0.197±0.006 | 0.026 | 0.023±0.0042 |
|  | Imported^b^ | 0.354 |  | 0.125 |  | 0.193 |  | 0.02 |  |
| Sumac | FSMS^a^ | 0.186 | 0.18±0.008 | 0.127 | 0.13±0.004 | 0.186 | 0.183±0.005 | 0.006 | 0.0065±0.0007 |
|  | FSMS^b^ | 0.174 |  | 0.133 |  | 0.179 |  | 0.007 |  |
|  | NFSMS^a^ | 0.323 | 0.299±0.035 | 0.14 | 0.1385±0.002 | 0.188 | 0.194±0.008 | 0.002 | 0.0045±0.0035 |
|  | NFSMS^b^ | 0.274 |  | 0.137 |  | 0.2 |  | 0.002 |  |
|  | Bulk^a^ | 0.316 | 0.313±0.004 | 0.104 | 0.12±0.022 | 0.176 | 0.184±0.011 | 0.0199 | 0.0205±0.0008 |
|  | Bulk^b^ | 0.31 |  | 0.136 |  | 0.191 |  | 0.0211 |  |
| Cumin | FSMS^a^ | 0.376 | 0.352±0.034 | 0.032 | 0.0305±0.002 | 0.18 | 0.183±0.004 | 0.003 | 0.005±0.003 |
|  | FSMS^b^ | 0.328 |  | 0.029 |  | 0.185 |  | 0.007 |  |
|  | NFSMS^a^ | 0.319 | 0.301±0.025 | 0.082 | 0.0755±0.009 | 0.152 | 0.174±0.03 | 0.01 | 0.011±0.001 |
|  | NFSMS^b^ | 0.283 |  | 0.069 |  | 0.195 |  | 0.012 |  |
|  | Bulk^a^ | 0.318 | 0.299±0.027 | 0.107 | 0.119±0.017 | 0.202 | 0.195±0.01 | 0.005 | 0.006±0 |
|  | Bulk^b^ | 0.28 |  | 0.132 |  | 0.188 |  | 0.006 |  |
|  | Imported^a^ | 0.337 | 0.346±0.013 | 0.153 | 0.139±0.019 | 0.204 | 0.186±0.026 | 0.008 | 0.009±0.001 |
|  | Imported^b^ | 0.355 |  | 0.125 |  | 0.167 |  | 0.01 |  |
| Sesame | FSMS^a^ | 0.189 | 0.207±0.25 | 0.136 | 0.134±0.002 | 0.198 | 0.187±0.016 | 0.008 | 0.011±0.004 |
|  | FSMS^b^ | 0.225 |  | 0.132 |  | 0.176 |  | 0.013 |  |
|  | NFSMS^a^ | 0.243 | 0.243±0 | 0.144 | 0.122±0.032 | 0.187 | 0.183±0.006 | 0.028 | 0.027±0.0028 |
|  | NFSMS^b^ | 0.242 |  | 0.099 |  | 0.179 |  | 0.024 |  |
|  | Bulk^a^ | 0.273 | 0.283±0.013 | 0.138 | 0.119±0.028 | 0.119 | 0.125±0.008 | 0.011 | 0.01±0.0014 |
|  | Bulk^b^ | 0.292 |  | 0.099 |  | 0.131 |  | 0.009 |  |
| Paprika | FSMS^a^ | 0.219 | 0.22±0 | 0.14 | 0.113±0.039 | 0.116 | 0.112±0.006 | 0.004 | 0.005±0 |
|  | FSMS^b^ | 0.22 |  | 0.085 |  | 0.107 |  | 0.005 |  |
|  | NFSMS^a^ | 0.487 | 0.482±0.007 | 0.062 | 0.09±0.04 | 0.041 | 0.048±0.009 | 0.003 | 0.003±0 |
|  | NFSMS^b^ | 0.477 |  | 0.118 |  | 0.054 |  | 0.003 |  |
|  | Bulk^a^ | 0.483 | 0.486±0.004 | 0.06 | 0.054±0.008 | 0.13 | 0.126±0.006 | 0.005 | 0.005±0 |
|  | Bulk^b^ | 0.489 |  | 0.048 |  | 0.121 |  | 0.004 |  |
|  | Imported^a^ | 0.311 | 0.33±0.027 | 0.004 | 0.004±0 | 0.058 | 0.065±0.009 | 0.005 | 0.005±0 |
|  | Imported^b^ | 0.349 |  | 0.004 |  | 0.071 |  | 0.004 |  |
| Dried Mint | FSMS^a^ | 0.298 | 0.292±0.008 | 0.058 | 0.051±0.01 | 0.026 | 0.018±0.011 | 0.021 | 0.017±0.006 |
|  | FSMS^b^ | 0.286 |  | 0.044 |  | 0.01 |  | 0.012 |  |
|  | NFSMS^a^ | 0.368 | 0.366±0.003 | 0.043 | 0.046±0.004 | 0.068 | 0.061±0.011 | 0.012 | 0.015±0.004 |
|  | NFSMS^b^ | 0.364 |  | 0.049 |  | 0.053 |  | 0.017 |  |
|  | Bulk^a^ | 0.364 | 0.38±0.023 | 0.047 | 0.041±0.009 | 0.075 | 0.084±0.012 | 0.015 | 0.018±0.004 |
|  | Bulk^b^ | 0.396 |  | 0.034 |  | 0.092 |  | 0.021 |  |
| White Pepper | FSMS^a^ | 0.23 | 0.228±0.004 | 0.004 | 0.004±0 | 0.079 | 0.067±0.018 | 0.009 | 0.01±0.001 |
|  | FSMS^b^ | 0.225 |  | 0.004 |  | 0.054 |  | 0.011 |  |
|  | NFSMS^a^ | 0.323 | 0.331±0.011 | 0.005 | 0.004±0 | 0.031 | 0.038±0.01 | 0.018 | 0.019±0.001 |
|  | NFSMS^b^ | 0.339 |  | 0.004 |  | 0.045 |  | 0.02 |  |
|  | Bulk^a^ | 0.377 | 0.381±0.006 | 0.092 | 0.091±0.002 | 0.018 | 0.017±0.001 | 0.012 | 0.015±0.004 |
|  | Bulk^b^ | 0.385 |  | 0.089 |  | 0.016 |  | 0.017 |  |
|  | Imported^a^ | 0.273 | 0.274±0.001 | 0.002 | 0.002±0 | 0.043 | 0.047±0.005 | 0.019 | 0.015±0.006 |
|  | Imported^b^ | 0.275 |  | 0.002 |  | 0.05 |  | 0.01 |  |
| Thyme Mix | FSMS^a^ | 0.243 | 0.243±0 | 0.062 | 0.061±0.002 | 0.044 | 0.046±0.003 | 0.018 | 0.014±0.006 |
|  | FSMS^b^ | 0.242 |  | 0.059 |  | 0.048 |  | 0.01 |  |
|  | NFSMS^a^ | 0.29 | 0.275±0.022 | 0.008 | 0.01±0.002 | 0.059 | 0.06±0.001 | 0.005 | 0.011±0.008 |
|  | NFSMS^b^ | 0.259 |  | 0.011 |  | 0.061 |  | 0.017 |  |
|  | Bulk^a^ | 0.289 | 0.281±0.011 | 0.38 | 0.345±0.049 | 0.079 | 0.071±0.011 | 0.015 | 0.017±0.002 |
|  | Bulk^b^ | 0.273 |  | 0.31 |  | 0.063 |  | 0.018 |  |
| Thyme | FSMS^a^ | 0.258 | 0.244±0.02 | 0.021 | 0.022±0.001 | 0.222 | 0.236±0.02 | 0.03 | 0.025±0.007 |
|  | FSMS^b^ | 0.23 |  | 0.023 |  | 0.25 |  | 0.02 |  |
|  | NFSMS^a^ | 0.315 | 0.299±0.023 | 0.008 | 0.008±0 | 0.041 | 0.048±0.009 | 0.023 | 0.022±0.001 |
|  | NFSMS^b^ | 0.283 |  | 0.007 |  | 0.054 |  | 0.021 |  |
|  | Bulk^a^ | 0.269 | 0.258±0.016 | 0.023 | 0.025±0.002 | 0.041 | 0.045±0.006 | 0.038 | 0.033±0.008 |
|  | Bulk^b^ | 0.247 |  | 0.026 |  | 0.049 |  | 0.027 |  |
|  | Imported^a^ | 0.285 | 0.277±0.011 | 0.046 | 0.046±0 | 0.017 | 0.019±0.002 | 0.026 | 0.029±0.004 |
|  | Imported^b^ | 0.269 |  | 0.046 |  | 0.2 |  | 0.032 |  |
| Red Chilli | FSMS^a^ | 0.227 | 0.223±0.006 | 0.062 | 0.06±0.003 | 0.022 | 0.025±0.004 | 0.002 | 0.004±0.002 |
|  | FSMS^b^ | 0.219 |  | 0.058 |  | 0.028 |  | 0.005 |  |
|  | NFSMS^a^ | 0.053 | 0.052±0 | 0.2 | 0.204±0.006 | 0.1 | 0.096±0.006 | 0.003 | 0.005±0.003 |
|  | NFSMS^b^ | 0.052 |  | 0.208 |  | 0.092 |  | 0.007 |  |
|  | Bulk^a^ | 0.433 | 0.421±0.018 | 0.103 | 0.104±0.001 | 0.097 | 0.092±0.008 | 0.007 | 0.008±0.001 |
|  | Bulk^b^ | 0.408 |  | 0.105 |  | 0.086 |  | 0.009 |  |
|  | Imported^a^ | 0.383 | 0.375±0.011 | 0.003 | 0.003±0 | 0.04 | 0.036±0.006 | 0.003 | 0.005±0.003 |
|  | Imported | 0.367 |  | 0.003 |  | 0.31 |  | 0.007 |  |
| Oregano | FSMS^a^ | 0.474 | 0.467±0.01 | 0.025 | 0.026±0 | 0.234 | 0.242±0.011 | 0.024 | 0.026±0.002 |
|  | FSMS^b^ | 0.46 |  | 0.026 |  | 0.25 |  | 0.027 |  |
|  | NFSMS^a^ | 0.283 | 0.285±0.003 | 0.067 | 0.065±0.004 | 0.117 | 0.108±0.013 | 0.027 | 0.02±0.01 |
|  | NFSMS^b^ | 0.287 |  | 0.062 |  | 0.098 |  | 0.013 |  |
|  | Bulk^a^ | 0.24 | 0.244±0.006 | 0.099 | 0.095±0.006 | 0.056 | 0.05±0.008 | 0.026 | 0.025±0.002 |
|  | Bulk^b^ | 0.248 |  | 0.091 |  | 0.044 |  | 0.023 |  |
|  | Imported^a^ | 0.353 | 0.361±0.011 | 0.049 | 0.049±0 | 0.137 | 0.126±0.016 | 0.028 | 0.023±0.008 |
|  | Imported^b^ | 0.368 |  | 0.048 |  | 0.155 |  | 0.017 |  |
| Garlic | FSMS^a^ | 0.212 | 0.215±0.004 | 0.024 | 0.023±0.002 | 0.021 | 0.03±0.013 | 0.004 | 0.005±0.001 |
|  | FSMS^b^ | 0.218 |  | 0.021 |  | 0.039 |  | 0.006 |  |
|  | NFSMS^a^ | 0.209 | 0.211±0.003 | 0.04 | 0.036±0.006 | 0.029 | 0.03±0.001 | 0.003 | 0.005±0.003 |
|  | NFSMS^b^ | 0.213 |  | 0.032 |  | 0.031 |  | 0.007 |  |
|  | Bulk^a^ | 0.247 | 0.244±0.004 | 0.023 | 0.023±0 | 0.061 | 0.069±0.011 | 0.014 | 0.009±0.007 |
|  | Bulk^b^ | 0.241 |  | 0.023 |  | 0.077 |  | 0.004 |  |
|  | Imported^a^ | 0.259 | 0.256±0.004 | 0.071 | 0.076±0.006 | 0.056 | 0.063±0.009 | 0.007 | 0.006±0.002 |
|  | Imported^b^ | 0.253 |  | 0.08 |  | 0.069 |  | 0.004 |  |

a: first collection.

b: second collection.
